# Synthesis of Branched Cyclo-Olefin Copolymers Using Neutral *α*-Sulfonate-*β*-Diimine Nickel Catalyst

**DOI:** 10.3390/molecules30010157

**Published:** 2025-01-03

**Authors:** Donghui Li, Lixia Pei, Wenbo Du, Xieyi Xiao, Heng Gao, Handou Zheng, Haiyang Gao

**Affiliations:** 1 School of Materials Science and Engineering, PCFM Lab, GD HPPC Lab, Sun Yat-sen University, Guangzhou 510275, China; ldonghui2017@163.com (D.L.); dwb5310@163.com (W.D.); xiaoxy53@mail2.sysu.edu.cn (X.X.); gaoheng16@163.com (H.G.); zhenghdou@mail.sysu.edu.cn (H.Z.); 2 School of Chemistry and Chemical Engineering, South China University of Technology, Guangzhou 510641, China

**Keywords:** cyclo-olefin copolymer, norbornene, ethylene, nickel catalyst, copolymerization

## Abstract

The homopolymerization of norbornene and the copolymerization of norbornene and ethylene were carried out using the neutral *α*-sulfonate-*β*-diimine nickel catalyst **SD-Ni**. The neutral *α*-sulfonate-*β*-diimine catalyst is highly active in the homopolymerization of norbornene, producing vinyl-addition polynorbornene (PNB) with a high molecular weight. The copolymerization of norbornene (NB) and ethylene (E) using the catalyst **SD-Ni** was also investigated. The *α*-sulfonate-*β*-diimine catalyst **SD-Ni** shows distinctive catalytic copolymerization properties to produce high-molecular-weight E-NB copolymers with low norbornene incorporation. Importantly, microstructure analyses confirm that the resultant E-NB copolymers are branched cyclo-olefin copolymers (COCs) with branched polyethylene units.

## 1. Introduction

Polymers of cyclic olefins such as norbornene (NB) with rigid and saturated cyclic units on polymer chains have garnered widespread attention due to their attractive properties such as excellent chemical resistance, good UV resistance, low dielectric constant, excellent transparency, and a large refractive index [1,2]. However, polynorbornene (PNB) produced by vinylic addition polymerization is a very brittle material at room temperature, which largely limits its application. Furthermore, the PNB material also exhibits very poor melt processability because its glass transition temperature (*T_g_*) is very high and near to its decomposition temperature [3].

The incorporation of flexible units into the rigid PNB chain to produce cyclo-olefin copolymers (COCs) by the copolymerization of norbornene with ethylene, *α*-olefins, and styrene comonomers is an effective strategy to improve toughness and decrease glass transition temperature (*T_g_*) [4,5,6]. Because ethylene is a cheap and abundant monomer, the copolymerization of norbornene and ethylene has received considerable attention and has been successfully commercialized [7,8]. Since the first copolymerization of norbornene and ethylene using zirconocene catalysts [9], various catalysts including early transition metal group IV catalysts and late transition metal group VIII catalysts have been mainly developed for the synthesis of COCs by the copolymerization of norbornene with ethylene [10,11,12].

**Figure 1:**
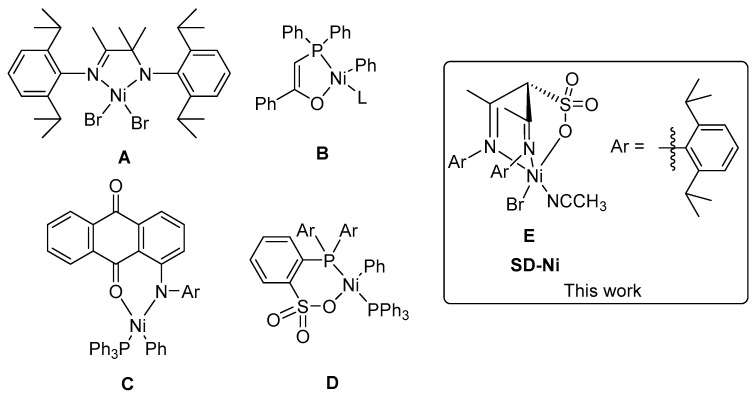
Nickel complexes for the copolymerization of norbornene with ethylene.

Compared to early transition metal group IV catalysts including metallocene [8], half-sandwich catalysts, [13,14] constrained-geometry group catalysts (CGCs) [1], and non-metallocene [15,16,17,18,19,20], late transition metal, nickel, and palladium catalysts are more challenging for the copolymerization of norbornene and ethylene [21,22,23,24]. Although late transition metal, nickel, and palladium catalysts are highly active for the homopolymerization of norbornene and ethylene, they often produce low-molecular-weight PNB with a vinyl chain end for norbornene polymerization in the presence of ethylene because ethylene is a very efficient chain transfer agent [25,26]. Only a few catalytic systems can afford high-molecular-weight alternating or block copolymers [26,27,28,29,30,31]. For example, our group reports the synthesis of polyethylene-*block*-polynorbornene (PE-*b*-PNB) copolymers using an amine–imine nickel catalyst (**A** in Figure 1) by sequential monomer addition in a living fashion [26]. Neutral phosphorylate nickel complexes (**B** in Figure 1) are efficient catalysts for the copolymerization of norbornene with ethylene to produce an essentially alternating copolymer [27,28]. Anilinoanthraquinone nickel catalysts (**C** in Figure 1) can catalyze the copolymerization of norbornene and ethylene with high activity to generate random copolymers with high norbornene content of 63.7–92.2 mol% [29]. Phosphine sulfonate nickel catalysts (**D** in Figure 1) also show good activity for the copolymerization of norbornene and ethylene to produce copolymers with norbornene incorporation of up to 10.1 mol% [30,31].

From the perspective of the microstructure of the chain, COCs with different sequences such as alternating, random, and block copolymers have been synthesized but the incorporated ethylene units are completely linear [20,26,32,33]. The branched ethylene units have never been reported to the best of our knowledge although a branched structure can further improve toughness and decrease the glass transition temperatures of the materials. In theory, branched COCs are not prepared by early transition metal group IV catalysts but are probably synthesized by late transition metal catalysts based on the chain-walking mechanism of ethylene polymerization [34,35,36].

In this paper, we initially report the synthesis of branched cyclo-olefin copolymers by the copolymerization of norbornene and ethylene using a neutral *α*-sulfonate-*β*-diimine nickel catalyst. In this catalytic system, ethylene can be successively inserted into the main chain and does not play the role of a chain transfer agent. The chain-walking process leads to the formation of branched ethylene units incorporated into the PNB chain.

## 2. Results and Discussion

The neutral *α*-sulfonate-*β*-diimine nickel complex has a tridentate molecular structure (**E** in Figure 1), and has been previously synthesized by our group [37]. We have also reported the ethylene polymerization properties of the neutral *α*-sulfonate-*β*-diimine nickel complex [37]. Herein, we further studied norbornene homopolymerization before the investigation of the copolymerization of norbornene and ethylene.

When the neutral *α*-sulfonate-*β*-diimine nickel complex **SD-Ni** was employed in norbornene polymerization in the absence of any cocatalysts (entry 1 in Table 1), no catalytic activity was observed. Therefore, the cocatalyst is essential to activate the nickel complex for norbornene polymerization. Methylaluminoxane (MAO) was herein chosen as a cocatalyst for activating the nickel complex **SD-Ni**, and the Al/Ni ratio effects on norbornene polymerization were first studied.

As shown in Table 1, the catalytic activity of **SD-Ni** for norbornene polymerization increases, and then decreases as the Al/Ni ratio increases from 1000 to 5000. An optimum Al/Ni ratio is 4000 for norbornene polymerization, which is higher than that for ethylene polymerization (Al/Ni = 1000). This may be because norbornene monomer is bulkier than ethylene monomer. Moreover, the Al/Ni ratio has an influence on the molecular weight of the produced polynorbornene (PNB). With an increase in the Al/Ni ratio, the molecular weights of PNBs decreased consistently, strongly indicating that chain transfer to aluminum cocatalyst takes place [38,39].

The reaction temperature also affected the catalytic activities and PNB molecular weight; therefore, the effects of temperature are further studied at the fixed Al/Ni ratio of 4000. As shown in Table 1, the catalytic activity of **SD-Ni** increases for norbornene polymerization, and then decreases as the polymerization temperature increases from 0 to 100 °C. At 80 °C, **SD-Ni** exhibits the highest activity of 3.41 × 10^6^ g PNB/(mol Ni h). Furthermore, polymerization temperature has an important influence on the molecular weight of the produced polynorbornene. As temperature increases from 0 to 25 °C, the PNB molecular weight increases. This is because increased temperature can promote the coordination and insertion of bulky norbornene at low temperatures. As temperature increases from 25 to 100 °C, the PNB molecular weight decreases due to the acceleration of the chain transfer to the aluminum cocatalyst at high temperatures [40,41].

**Figure 2:**
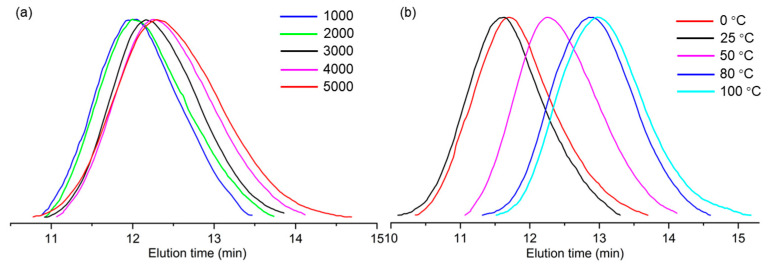
GPC curves of PNB samples prepared by **SD-Ni** under different Al/Ni ratios (**a**) and different temperatures (**b**).

All of the produced PNBs catalyzed by the catalyst **SD-Ni** are soluble in organic solvents such as chlorobenzene and *o*-dichlorobenzene. High-temperature gel permeation chromatography (GPC) was used to determine the molecular weight and distribution of the produced PNBs using 1,2,4-trichlorobenzene as an eluent at 150 °C (Figure 2). The produced PNBs are high-molecular-weight polymers, and the highest *M*_w_ is up to 1339.7 kg/mol. The molecular weight distributions are normal (*M*_w_/*M*_n_ = 1.5~2.0).

**Figure 3:**
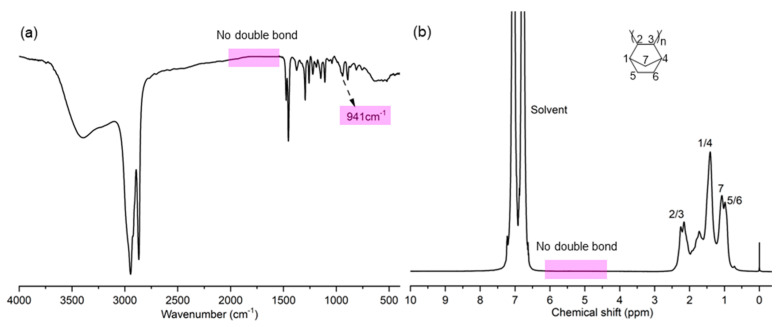
FT-IR (**a**) and ^1^H NMR (**b**) spectra of PNB (entry 5 in Table 1).

**Figure 4:**
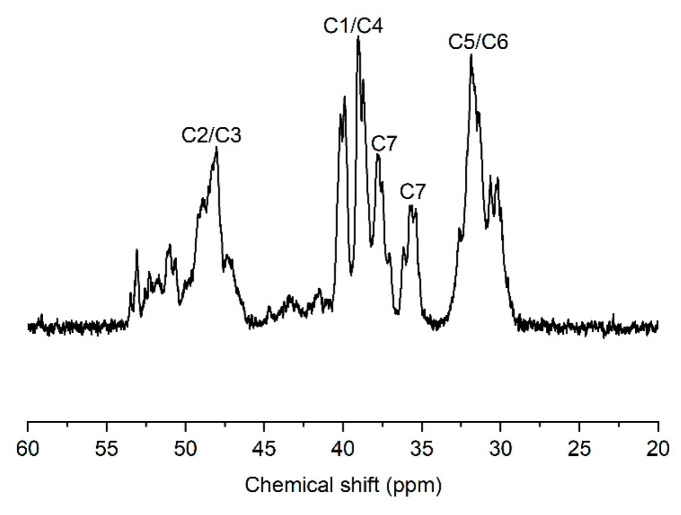
^13^C NMR spectrum of PNB (entry 5 in Table 1).

The microstructure of the produced PNBs was further characterized. The FT-IR spectrum (Figure 3a) reveals no traces corresponding to double bonds observed at 1680~1620, 966, and 735 cm^−1^, and the appearance of vibration bands of the bicyclic structure of norbornene at 941 cm^−1^. The ^1^H NMR spectrum (Figure 3b) also shows no double bond at 5.0~6.0 ppm, indicating that the produced PNBs are vinyl-addition polymers [42,43,44]. The ^13^C NMR spectrum further confirms vinyl-addition PNB, showing that several resonances are present at 28~55 ppm (Figure 4). The resonances at 29.4~33.0, 35.5~38.5, 38.8~42.8, and 46.1~54.0 ppm are assigned to C_5_ and C_6_, C_7_, C_1_ and C_4_, C_2_ and C_3_, respectively [30,45,46,47,48].

**Figure 5:**
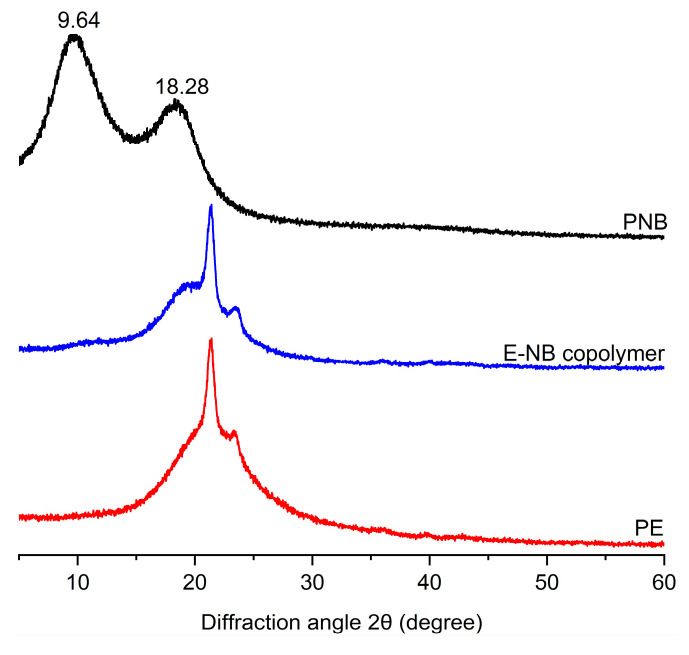
WAXD curves of PNB (entry 5 in Table 1); E-NB copolymer (entry 4 in Table 2); and PE (entry 1 in Table 2).

The wide-angle X-ray diffraction (WAXD) pattern of PNB (Figure 5) shows that there are two broad and diffuse diffraction peaks at near 2θ values of 9.64° and 18.28°, indicating that the PNB is an amorphous polymer with long-range disorder [49,50]. The glass transition temperature (*T*_g_) of the PNB was determined by DSC testing. No well-defined endothermic signal is determined in the temperature range from −20 to 300 °C because the glass transition temperature of PNB is close to its decomposition temperature.

**Figure 6:**
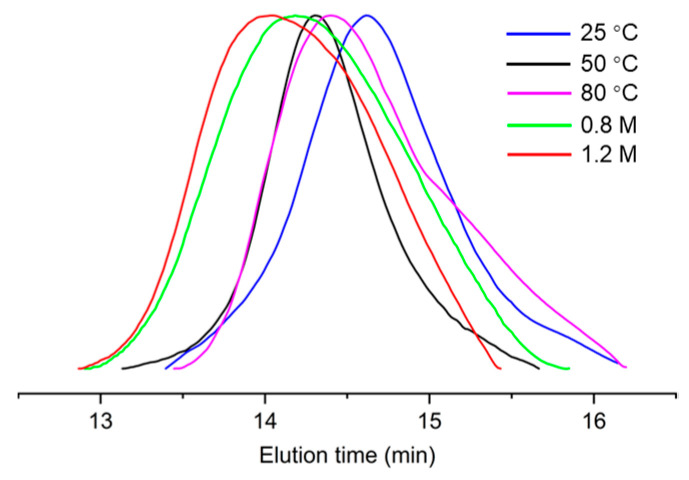
GPC curves of E-NB copolymers prepared by **SD-Ni**.

We have previously studied the ethylene polymerization properties of the neutral *α*-sulfonate-*β*-diimine nickel complex **SD-Ni** [37]. Inspired by its high activity for the homopolymerization of norbornene and ethylene, we further carried out the copolymerization of norbornene and ethylene using the catalyst **SD-Ni** and the copolymerization results are summarized in Table 2. GPC analyses (Figure 6) of the copolymerization products show that their distributions remain below 2.0 and exhibit a unimodal distribution, indicating that copolymerizations truly occur and the products are copolymers rather than a blend of homopolymers.

**Figure 7:**
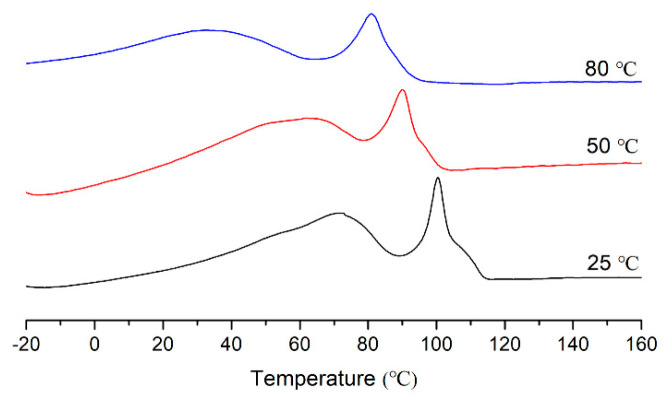
DSC curves of E-NB copolymers produced at different temperatures (entries 2–4 in Table 2).

The effects of polymerization temperature on the copolymerization of norbornene and ethylene were studied at the fixed norbornene concentration (0.4 M). By increasing temperature from 25 to 80 °C, the copolymerization activity increases and then decreases. At 50 °C, copolymerization activity reaches a maximum value of 24.2 × 10^3^ g COC/(mol Ni h). Compared to the homopolymerization activity of ethylene (entry 1 in Table 2), the copolymerization activity decreases because the insertion of bulky norbornene is more difficult than ethylene monomer [31]. Additionally, the *α*-sulfonate-*β*-diimine nickel showed comparable copolymerization activity compared to previously reported late transition metal catalysts [27,28,29,30]. The influence of polymerization temperature on copolymer molecular weight also shows the same tendency. The copolymer produced at 50 °C has the highest molecular weight. Moreover, increasing temperature leads to an increase in the incorporation of norbornene; the produced E-NB copolymers contain low norbornene incorporation (<10 mol%), which supports the conclusion that the insertion of bulky norbornene is slower than ethylene monomer. Because of low norbornene incorporation, the produced E-NB copolymers display broad melting temperatures due to long ethylene sequences and branched structures. A clear trend is that melting temperatures decrease as polymerization temperatures increase due to greater norbornene incorporation and higher branch density (Figure 7).

**Figure 8:**
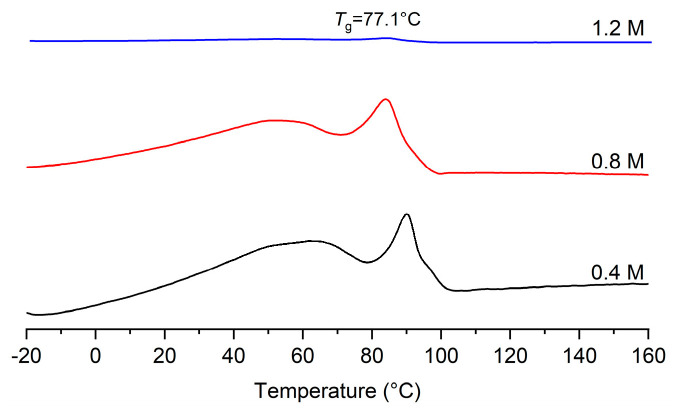
DSC curves of E-NB copolymers produced at different norbornene concentrations (entries 3, 5, 6 in Table 2).

The effects of norbornene feed on the copolymerization of norbornene and ethylene were studied at 50 °C. By increasing norbornene concentration from 0.4 to 1.2 M, the copolymerization activity decreases gradually. This observation also supports the idea that ethylene is inserted more favorably than bulky norbornene by the nickel catalyst **SD-Ni**. It is noteworthy that the copolymer molecular weight increases with increasing norbornene concentration, strongly indicating that the chain transfer reaction to the ethylene monomer is favorable during copolymerization. This result is consistent with the homopolymerization result of norbornene mentioned above. Furthermore, increasing norbornene concentration leads to the increasing incorporation of norbornene. At a norbornene concentration of 1.2 M, the incorporation of norbornene reaches 22.1 mol%. As a result, the obtained E-NB copolymer has a *T*_g_ of 77.1 °C but not a *T*_m_ (Figure 8).

Additionally, we note that the melting temperatures of the copolymers are lower than those of previously reported E-NB copolymers produced by other catalysts. In phosphine-sulfonate nickel catalysts, the obtained E-NB copolymers with 7 mol% and 4 mol% incorporations of NB show melting temperatures of 90 °C and 108 °C [30]. However, the E-NB copolymers with the same NB incorporations produced by our nickel catalyst **SD-Ni** display lower and broader melting temperatures (79.5 °C and 90.1 °C), which are ascribed to branched polyethylene units. Furthermore, the E-NB copolymer produced at 0.8 M (entry 5 in Table 2) has a lower melting temperature than the E-NB copolymer produced at 80 °C although the former has higher NB incorporation (10.6 mol%) than the latter (7.2 mol%). This is a result of the high branching density of polyethylene, as high temperatures favor chain walking.

**Figure 9:**
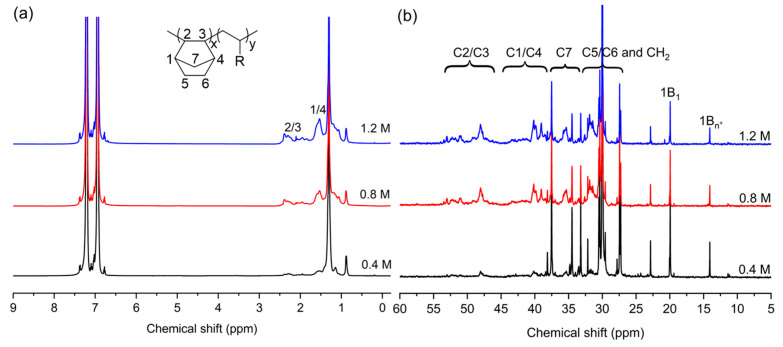
^1^H NMR (**a**) and ^13^C NMR (**b**) spectra of E-NB copolymers produced at different concentrations (entries 3, 5, 6 in Table 2).

Microstructures of the E-NB copolymers were further investigated by ^1^H, ^13^C NMR spectroscopies. As shown in Figure 9a, the ^1^H NMR spectrum of E-NB copolymers is more complex than previously reported spectra because of the presence of various branchings. The calculation of the branching density of the PE blocks is difficult because the signals of CH and CH_2_ of norbornene units and PE units overlap. Therefore, the branching density of the PE blocks can be speculated according to the PE produced by the homopolymerization of ethylene (30~50/1000C) [37]. As shown in Figure 9b, the ^13^C NMR spectrum of the copolymer containing a norbornene incorporation of 7.2% obtained at a norbornene concentration of 0.4 M primarily displays signals of branched polyethylene, and several weak peaks are observed and assigned to norbornene units. Based on the previous assignment of polyethylene [36], the peak at 19.98 ppm is assigned to the methyl group, and the peak at 14.60 ppm is assigned to the long-chain branching. Therefore, the obtained E-NB copolymers are branched COCs. To the best of our knowledge, this is the first report on branched COCs with branched ethylene units. The formation of branched COCs can be ascribed to two reasons. On one hand, the neutral *α*-sulfonate-*β*-diimine nickel catalyst displays distinctive copolymerization properties. In this catalytic system, the ethylene monomer is favorably inserted rather than a norbornene monomer and does not act as a chain transfer agent, which is different from previously reported nickel catalysts [25,26]. On the other hand, the neutral *α*-sulfonate-*β*-diimine nickel catalyst has good chain-walking ability, which assures the formation of branched ethylene units [37].

In the ^13^C NMR spectra of E-NB copolymers with high NB incorporation prepared at high norbornene concentrations (Figure 9b), signals of polynorbornene obviously heighten but resonances of branching polyethylene weaken. According to previous reports and assignments [51,52,53,54], the copolymers have random structures. The ^13^C NMR analysis is in strong agreement with the DSC analysis. Wide-angle X-ray diffraction analysis (WAXD) (Figure 5) shows that the obtained E–NB copolymer is amorphous and has low stereoregularity. Compared to the WAXD patterns of homopolymers (PE and PNB), the obtained E–NB copolymer exhibits broad halos.

Clearly, branched polyethylene units originate from chain walking, which is a common phenomenon during nickel- and palladium-catalyzed ethylene polymerization. Our experimental results also show that ethylene is more favorably inserted into the *α*-sulfonate-*β*-diimine nickel catalyst than bulky norbornene monomer because of the low electrophilicity of the neutral nickel center and bulky steric hindrance of the catalyst. Resultantly, the continuous insertion of ethylene monomer is more conducive to the formation of branched PE units through the chain-walking mechanism during the copolymerization of ethylene and norbornene.

## 3. Materials and Methods

### 3.1. General Procedures

All operations with air- and moisture-sensitive compounds were conducted under a dried and purified nitrogen atmosphere, utilizing standard vacuum-line and Schlenk techniques, or a glovebox.

### 3.2. Materials

Toluene was distilled from Na/K alloy under nitrogen before use. Norbornene was purchased from HWRK Chem Co. Ltd. (Beijing, China) and purified by distillation over CaH_2_ at a reduced pressure. Methylaluminoxane (MAO, 30 wt% in toluene) was purchased from Akzo-Nobel (Amsterdam, The Netherlands). Other commercially available reagents were purchased and used without purification. The *α*-sulfonate-*β*-diimine nickel was prepared according to the our previously reported literature [37]. The 2.672 g PNB sample (entry 5 in Table 1) was further purified by extraction by 10% HCl/ethanol solution and then dried to a constant weight before FT-IR and ^1^H NMR analysis.

### 3.3. Measurements

^1^H, ^13^C NMR spectra of polymers were carried out on a Bruker 500 MHz at 120 °C with a 74° flip angle, an acquisition time of 1.5 s, and a delay of 4.0 s. Polymer solutions were prepared in *o*-C_6_H_4_Cl_2_/*o*-C_6_D_4_Cl_2_ (50% *v*/*v*) in a 10 mm sample tube. DSC analyses were conducted with a PerkinElmer DSC-4000 system. During testing, mechanical refrigeration was used with nitrogen protection, and the mass of solid samples ranged from 3.0 to 6.0 mg. The DSC curves were recorded as second heating curves from −20 to 300 °C at a heating/cooling rate of 10 °C/min. FT-IR spectra were recorded on a Nicolet Nexus 670 FT-IR spectrometer (Thermo Nicolet, Waltham, MA, USA). The KBr pellet method was employed, with a measurement range of 4000–400 cm^−1^ and a resolution of 2 cm^−1^. GPC analysis of the molecular weights and molecular weight distributions (*M*_w_/*M*_n_) of the polymers at 150 °C was performed on a PL-GPC 220 high-temperature chromatograph equipped with two GPC columns (USA PLgel 10 μm MIXED-B) and a differential refractive-index detector. 1,2,4-Trichlorobenzene (TCB) was used as the eluent at a flow rate of 1.0 mL/min. An appropriate amount of polymer was weighed and dissolved in TCB to prepare a 1 g/mL solution. The solution was heated on a preheating stage at 150 °C for 4 h until the polymer fully dissolved. The dissolved solution was filtered through a polyimide membrane using a specialized filter and then tested using an automatic sampling system. The molecular weight data were analyzed using narrow polystyrene standards (Polymer Laboratories, Long Beach, CA, USA) and were corrected for polymer samples by universal calibration. The wide-angle X-ray diffraction (WAXD) curve of the polymer powder was obtained using a D-Max-2200 VPC powder X-ray diffractometer (RIGAKU Corporation, Tokyo, Japen). The polymer was loaded into the sample holder groove, ensuring that the polymer powder was evenly distributed without significant accumulation or voids. Clean weighing paper was used to evenly press the powder, gradually compacting it to fit tightly within the groove. CuK_a_ radiation (λ = 0.154 nm, 40 kV) was used as the X-ray source. The diffraction angle was scanned over a range of 5–60° at a scanning rate of 10°/min.

### 3.4. Norbornene Homopolymerization

In a typical polymerization procedure, a 50 mL Schlenk round-bottom flask was preheated at 150 °C for 2 h. The required amounts of MAO, toluene, and a solution of norbornene were added to the flask under a nitrogen atmosphere with continuous stirring. Once the reaction temperature was stabilized, the system was stirred for an additional 5 min before injecting the desired amount of nickel complex into the toluene. After the norbornene polymerization had proceeded for the specified time, the reaction was carefully quenched with a 10% HCl/ethanol solution. The resulting precipitated polymers were collected by filtration, washed several times with ethanol, and dried at 50 °C to a constant weight.

### 3.5. Copolymerization of Norbornene with Ethylene

A mechanically stirred 100 mL Parr reactor was dried at 150 °C and vacuumed for 2 h. The autoclave was pressurized to 2 atm pressure of ethylene and vented three times. The required amounts of MAO solution, norbornene, and toluene were added into the autoclave at the desired polymerization temperature. The system was maintained by continuously stirring for 5 min, and then 4 mL solution of nickel complex in toluene was charged into the autoclave. The ethylene pressure was raised to the specified value, and the reaction was carried out for a certain time. The reaction temperature was controlled by means of a heater or cooler and was monitored by an internal thermocouple. Polymerization was terminated by a mixture of 10% HCl/ethanol solution after releasing the ethylene pressure. The resulting precipitated polymers were collected by filtration, washed several times with ethanol, and dried at 50 °C to a constant weight.

## 4. Conclusions

The neutral *α*-sulfonate-*β*-diimine catalyst is highly active in the homopolymerization of norbornene, producing ultra-high-molecular-weight PNB (*M_w_* = 1339.7 kg/mol). Under optimum conditions of Al/Ni of 4000 and 80 °C, the highest activity of 3.41 × 10^6^ g PNB/(mol Ni h) is realized. For the copolymerizations of norbornene and ethylene, the *α*-sulfonate-*β*-diimine catalyst **SD-Ni** shows distinctive catalytic properties to produce E-NB copolymers containing low levels of norbornene incorporation. The incorporation of norbornene can be adjusted by the polymerization temperature and the norbornene feed. The microstructure analyses confirm that the resultant E-NB copolymers are branched COCs with branched polyethylene units. Branched COCs have not been prepared previously by other catalysts, and their properties will be further studied in the future.

## Figures and Tables

**Table 1:** Norbornene homopolymerization results using **SD-Ni** *^a^*.

Entry	Al/Ni	T (°C)	Yield (g)	Act. *^b^*	*M*_n_ *^c^* (kg/mol)	*M*_w_ *^c^* (kg/mol)	*M*_w_/*M*_n_ *^c^*
1	0	50	-	-	-	-	-
2	1000	50	0.782	0.78	632.9	1025.4	1.62
3	2000	50	1.123	1.12	549.6	961.9	1.75
4	3000	50	1.402	1.40	489.6	788.3	1.61
5	4000	50	2.672	2.67	446.2	736.2	1.65
6	5000	50	2.396	2.40	388.8	734.9	1.89
7	4000	0	1.356	1.36	613.9	1141.9	1.86
8	4000	25	1.868	1.87	774.4	1339.7	1.73
9	4000	80	3.413	3.41	256.5	451.4	1.76
10	4000	100	3.083	3.08	194.7	375.7	1.93

*^a^ *Polymerization conditions: 2 μmol Ni, NB = 0.0638 mol, 20 mL toluene, reaction time 0.5 h. *^b^ *Activity, in unit of 10^6^ g PNB/(mol Ni h). *^c^ M*_w_ and *M*_w_/*M*_n_ were determined by high-temperature gel permeation chromatography (GPC).

**Table 2:** Copolymerization results of norbornene and ethylene using **SD-Ni** *^a^.*

Entry	T (°C)	[NB] (mol/L)	Yield (g)	Act. *^b^*	I_NB_ *^c^* (mol %)	*M*_w_ *^d^*	*M*_w_/*M*_n_ *^d^*	*T*_m_/*T*_g_ *^e^*
1	50	-	0.916	45.8	-	207.1	1.43	82.3, 103.5/-
2	25	0.4	0.353	17.6	2.3	32.2	1.53	70.3, 100.5/-
3	50	0.4	0.485	24.2	4.8	44.1	1.41	61.6, 90.1/-
4	80	0.4	0.213	10.7	7.2	39.8	1.61	40.5, 79.5/-
5	50	0.8	0.352	17.6	10.6	62.8	1.64	53.3, 84.3/-
6	50	1.2	0.285	14.3	22.1	74.1	1.52	-/77.1

*^a^ *Polymerization conditions: 5 μmol Ni, 5 atm ethylene, Al (MAO)/Ni = 1000, 40 mL toluene, reaction time 4 h. *^b^* Activity, in unit of 10^3^ g COC/(mol Ni h). *^c^* Determined by ^13^C NMR spectroscopy. *^d^ M*_w_ (kg/mol) and *M*_w_/*M*_n_ were determined by high-temperature gel permeation chromatography (GPC). *^e^* Determined by differential scanning calorimetry (DSC).

## Data Availability

The data presented in this study are available on request from the corresponding author.
